# High Soluble Endoglin Levels Do Not Induce Endothelial Dysfunction in Mouse Aorta

**DOI:** 10.1371/journal.pone.0119665

**Published:** 2015-03-13

**Authors:** Ivana Nemeckova, Agnieszka Serwadczak, Barbara Oujo, Katerina Jezkova, Jana Rathouska, Petra Fikrova, Michala Varejckova, Carmelo Bernabeu, Jose M. Lopez-Novoa, Stefan Chlopicki, Petr Nachtigal

**Affiliations:** 1 Department of Biological and Medical Sciences, Faculty of Pharmacy in Hradec Kralove, Charles University in Prague, Heyrovskeho 1203, Hradec Kralove, 500 05, Czech Republic; 2 Jagiellonian Centre for Experimental Therapeutics (JCET), Bobrzynskiego 14, 30-348, Krakow, Poland; 3 Department of Experimental Pharmacology, Chair of Pharmacology, Medical College Jagiellonian University, Grzegorzecka 16, 31–531 Krakow, Poland; 4 Renal and Cardiovascular Physiopathology Unit, Department of Physiology and Pharmacology, University of Salamanca, 37007 Salamanca, Spain; 5 Centro de Investigaciones Biologicas, Consejo Superior de Investigaciones Cientificas, CSIC, and Centro de Investigacion Biomedica en Red de Enfermedades Raras (CIBERER), 28040 Madrid, Spain; Medical Faculty, Otto-von-Guericke University Magdeburg, Medical Faculty, GERMANY

## Abstract

Increased levels of a soluble form of endoglin (sEng) circulating in plasma have been detected in various pathological conditions related to cardiovascular system. High concentration of sEng was also proposed to contribute to the development of endothelial dysfunction, but there is no direct evidence to support this hypothesis. Therefore, in the present work we analyzed whether high sEng levels induce endothelial dysfunction in aorta by using transgenic mice with high expression of human sEng. Transgenic mice with high expression of human sEng on CBAxC57Bl/6J background (*Sol-Eng* ^+^) and age-matched transgenic littermates that do not develop high levels of human soluble endoglin (control animals in this study) on chow diet were used. As expected, male and female *Sol-Eng* ^+^ transgenic mice showed higher levels of plasma concentrations of human sEng as well as increased blood arterial pressure, as compared to control animals. Functional analysis either *in vivo* or *ex vivo* in isolated aorta demonstrated that the endothelium-dependent vascular function was similar in *Sol-Eng* ^+^ and control mice. In addition, Western blot analysis showed no differences between *Sol-Eng* ^+^ and control mice in the protein expression levels of endoglin, endothelial NO-synthase (eNOS) and pro-inflammatory ICAM-1 and VCAM-1 from aorta. Our results demonstrate that high levels of soluble endoglin alone do not induce endothelial dysfunction in *Sol-Eng* ^+^ mice. However, these data do not rule out the possibility that soluble endoglin might contribute to alteration of endothelial function in combination with other risk factors related to cardiovascular disorders.

## INTRODUCTION

Endoglin/CD105 (Eng) is an accessory type III receptor for several members of the transforming growth factor-β (TGF-β) superfamily of proteins. This homodimeric, 180 kDa, transmembrane glycoprotein is considered to play an eminent role in hematopoiesis, angiogenesis, cardiovascular development and vascular tone [1]. Endoglin is expressed predominantly in endothelial cells, but it can also be detected in various other cells, including smooth muscle cells, mesenchymal and hematopoietic stem cells, monocytes/macrophages, placental syncytiotrophoblasts and fibroblasts [2].

In addition to membrane-bound endoglin, increased levels of a soluble form of endoglin (sEng) have been detected in plasma in various pathological conditions related to the cardiovascular system. Circulating sEng represents the NH_2_-terminal cleavage product of full-length membrane-bound endoglin [3] and was proposed to be cleaved from the intact membrane form of several cell types including endothelial cells and trophoblasts by matrix metalloproteinase-14 (MMP-14 or MT-1) [4,5].

There is a number of reports suggesting that soluble endoglin may be regarded as a biomarker of endothelial dysfunction, for example in pre-eclampsia [3], atherosclerosis [6,7], hypercholesterolemia [8], diabetes mellitus and hypertension [9] and chronic coronary artery disease [10]. Because in all of these pathologies the endothelial dysfunction plays an important role, it was also proposed that high levels of soluble endoglin might represent a hallmark of endothelial dysfunction contributing to the development of numerous cardiovascular diseases, including pre-eclampsia and atherosclerosis [11]. On the other hand, it was also demonstrated that soluble endoglin is able to modify TGF-β-dependent signaling in vascular endothelium [12]. Despite being related to several cardiovascular pathologies, it is still unclear whether soluble endoglin represents a mere biomarker or it is mechanistically involved in vascular pathology via e.g. induction of endothelial dysfunction.

The increased expression of cell adhesion molecules, the impairment of NO bioavailability and NO-dependent vasodilatation are the general hallmarks of endothelial dysfunction, the crucial step in the pathogenesis of atherosclerosis [13–15]. Interestingly, it was demonstrated that soluble endoglin increases the expression of cell adhesion molecules, the number of rolling leukocytes and impairs endothelial dependent vascular function [12]. Furthermore, we have recently observed that soluble endoglin impairs leukocyte rolling and binding to endothelium *“in vitro”* [16].

In order to study the pathogenic role of soluble circulating form of Eng, a transgenic mouse model expressing human soluble endoglin (*Sol-Eng* ^*+*^) has been recently generated. The *Sol-Eng* ^*+*^ mice exhibit a pre-eclampsia-like phenotype, including hypertension, small pup size, proteinuria and renal damage [5].

Considering a possible role of soluble endoglin in the development of systemic endothelial dysfunction, in the present work, we have assessed whether in *Sol-Eng* ^*+*^ mice the endothelial dysfunction in aorta can be detected, as compared to their transgenic littermates with low levels of soluble endoglin.

## MATERIALS AND METHODS

### Animals and study design

A mouse line that overexpresses human soluble endoglin (*Sol-Eng* ^*+*^) on the CBAxC57BL/6J background was generated at the Genetically Modified Organisms Generation Unit (University of Salamanca, Spain), as previously described [5]. Four to six month old *Sol-Eng* ^*+*^ male and female mice with high plasma levels of soluble endoglin and their age matched male and female littermates with low plasma levels of soluble endoglin (transgenic mice with low plasma levels of sEng used as control mice) were used. The animals were housed under a 12-h light cycle and constant temperature and humidity and had free access to tap water and a standard laboratory pellet diet.

All experiments were performed in accordance with the directive of the EEC (86/609/EEC) and the use of animals was approved by the Ethical Committee for the protection of animals against cruelty at Faculty of Pharmacy in Hradec Kralove, Charles University in Prague (Permit Number: 21558/2013–2), and the Bioethics Committee of the University of Salamanca (Permit Number: 006–201400038812).

All surgery procedures were carried out under ketamine/diazepam/atropine or ketamine/xylazine anesthesia, and all efforts were made to minimize the suffering of the animals.

### Analysis of soluble endoglin concentration in plasma

Blood was extracted using a tail tip and plasma levels of human soluble endoglin were determined by means of Human Endoglin/CD105 Quantikine ELISA Kit (R&D Systems, MN, USA) according to the instructions of the manufacturer.

### Blood Pressure and heart rate measurement

Basal Blood pressure (BP) was recorded in conscious mice with an automated multichannel system by using the tail-cuff method and a photoelectric sensor (Niprem 546; Cibertec, Spain). Animals were previously accustomed for several days and measures were collected at the same hour during at least 3 days, as previously described [17–19]. Acute changes in BP and heart rate (HR) after drug administration were measured in conscious, freely moving mice by radiotelemetry techniques as previously described [17]. In brief, after anesthesia of the animals with a mixture of ketamine 78 mg/Kg, diazepam 6 mg/Kg, and atropine 0.15 mg/Kg; i.p., the carotid artery of the mouse was accessed with a ventral midline incision, and cannulated with a catheter attached to a combination pressure transducer, transmitter and battery, all encapsulated in an implantable microminiaturized electronic monitor (PA-C20, Data Sciences International-DSI-; MN, USA). The skin was closed with staples and tissue adhesive, and topical antiseptic was applied. An analgesic, (buprenorphine 0.1 mg/Kg i.m., Buprex, Schering-Plough, Spain) was given at the end of the surgery. An antibiotic (cefazolin 25 mg/Kg, i.m., Normon, Spain) was administered at the time of the operation and twice daily during recovery. Approximately 1 mL of normal saline was subcutaneously injected into two or more abdominal sites to assure adequate postoperative hydration, and the animal was kept in a ventilated and warm environment for at least 24 h. Each animal was housed individually in a standard polypropylene cage in a 12:12-h light-dark cycle room, fed standard rodent chow, and given drinking water *ad libitum*. At least 13 days after recovery from the surgical procedures, the cage was placed over a radio receiver in a quiet environment, and repeated measurements of basal systolic (SAP) and diastolic (DAP) arterial pressure and HR were performed in each animal between 10:00 a.m. and 14:00 p.m., for at least 3 days, to ensure stable pressure record. Data was digitally recorded on a computer and results were calculated using the software provided by Data Sciences. To assess the acute effects of the substances to be tested, basal SAP and DAP and HR were recorded for 5 min. Then, animals were given the different substances by i.p. injection in 0.1 mL isotonic NaCl, and AP and HR were continuously recorded for 30 min. Injection of 0.1 mL of isotonic NaCl induced a transient increase in AP and HR that disappeared in 1–2 min and was similar in WT and *Sol-Eng* ^*+*^ mice; thus, indicating that the effects observed for the different substances are not due to animal manipulation or differential response to stress between mouse strains. Substances tested were: acetylcholine 1 μg/Kg, sodium nitroprusside (SNP) 2 mg/Kg, and the nitric oxide synthase inhibitor—L-NG-nitroarginine methyl ester (L-NAME) 50 mg/Kg.

### Analysis of vascular function in isolated mice aorta

Animals received an anesthetic (mixture of ketamine 100 mg/Kg and xylazine 16 mg/Kg; i.p.) overdose and the thoracic part of the aorta was quickly removed, washed in Krebs-Hanseleit buffer and carefully dissected from surrounding tissue. Isolated aorta was cut into 4 rings (each 3 mm long) and placed in organ chambers of the wire myograph (620M, Danish Myo Technology, Denmark). The rings were mounted between 2 pins attached to an isometric force transducer with continuous recording of tension (PowerLab, LabChart, ADI Instruments, Australia) and gassed with 95% O_2_ and 5% CO_2_. After an equilibration and heating (37°C) period of 30 min, the tension was stepwise increased to 10 mN for further stabilization for 30 min. The viability of the vessels was checked by KCl (30–60 mM). Aortic rings were pre-contracted with increasing concentrations of phenylephrine (PHE, 0.01–1 μM) or prostaglandin F2alpha (PGF2α, 0.1–10 μM), respectively, to obtain approximately 80% of KCl induced contraction. The endothelium-dependent relaxation was induced by cumulative concentrations of acetylcholine (Ach, 0.01–1 μM). Modulatory effect of NO production on contractility was determined by analyzing the PHE induced contractility before and after the administration of L-NAME (300 μM, incubation 20 min).

### Expression of endothelial dysfunction markers in isolated mice aorta by Western blot

Samples of aorta (n = 7 controls, n = 6 *Sol-Eng* ^*+*^) were homogenized in RIPA lysis buffer (Sigma-Aldrich, St. Louis, USA) with protease (SERVA Electrophoresis, Germany) and phosphatase inhibitors (Thermo Fisher Scientific Inc., IL, USA). Proteins were separated by sodium dodecyl sulfate-polyacrylamide gel electrophoresis (SDS-PAGE) and electrically transferred onto PVDF membrane (Milipore, NY, USA) using Trans-Blot SD Semi-Dry Electrophoretic Transfer Cell (Bio-Rad, CA, USA). The membranes were blocked for 1 hour with 5% non-fat dry milk in Tris buffered saline containing 0.1% Tween-20 at room temperature, and then incubated with following primary antibodies: goat polyclonal anti-endoglin (90–95 kDa; dilution 1:500; sc-19793, Santa Cruz Biotechnology, Inc., CA, USA), rabbit polyclonal anti-eNOS (140kDa; dilution 1:200; sc-654, Santa Cruz Biotechnology, Inc.), rabbit polyclonal anti-ICAM-1 (85–110 kDa; dilution 1:500; sc-1511-R, Santa Cruz Biotechnology, Inc.), goat polyclonal anti-VCAM-1 (140 kDa, dilution 1:500; AF643, R&D Systems). Equal loading of proteins onto the gel was confirmed by immunodetection of mouse monoclonal anti-GAPDH antibody (37 kDa; dilution 1:10,000; G8795, Sigma-Aldrich). As secondary antibodies HRP-conjugated goat anti-mouse IgG at 1:20,000 (A9917, Sigma-Aldrich), HRP-linked goat anti-rabbit IgG—(Fab)´2 at 1:2,000 (ab6112, Abcam, UK) and HRP-conjugated rabbit anti-goat IgG at 1:5,000 (A5420, Sigma-Aldrich) were used. Membranes were developed using enhanced chemiluminescent reagent (Thermo Fisher Scientific Inc.) and subsequently exposed to X-Ray films (Foma, Czech Republic). The immunoreactive bands were scanned by using an Epson Perfection V5000 Photo (EPSON Inc., CA, USA) and semiquantified using NIS-Elements software, version 4.0 (Laboratory Imaging, Czech Republic).

### Urinary nitrite excretion

Urine was obtained from individual mice housed in metabolic cages for 24 h. Urine was collected in graduated cylinders containing 100 μL of 0.1% sodium azide (to minimize bacterial growth) and 1 mL of mineral oil (to avoid evaporation). Urinary nitrite concentration was determined in plasma and urine by a modification of the Griess reaction, as described [20]. Briefly, 500 μL of sample were mixed with 250 μL of Griess reagent (1% sulfanilamide and 0.1% naphthyl ethylenediamine dihydrochloride, in 2.5% orthophosphoric acid; Sigma Aldrich) and incubated for 15 min at room temperature. Absorbance was measured at 560 nm and calibration was carried out using sodium nitrite. Urine creatinine concentrations were determined by a modification of Jaffé’s reaction method.

### Statistical analysis

The statistical analysis was performed by GraphPad Prism 6.0 software (GraphPad Software, Inc., CA, USA). All data are presented as mean ± S.E.M. Direct group-group comparisons were carried out using one or two way ANOVA, paired or unpaired t-test and Mann-Whitney test as adequate. P values of 0.05 or less were considered statistically significant.

## RESULTS

### Elevated levels of human soluble endoglin in plasma in *Sol-Eng* ^*+*^ mice

**Fig 1:**
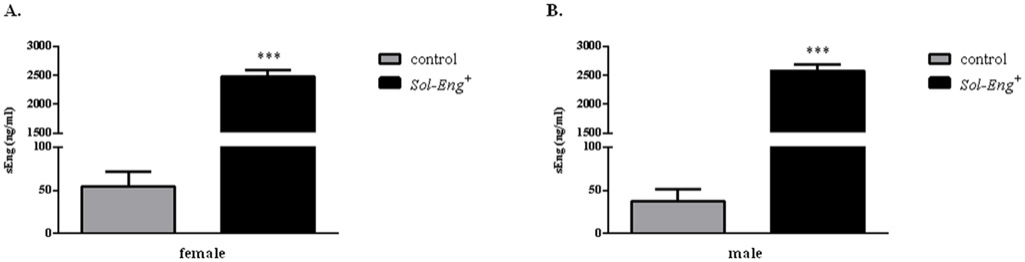
Concentrations of human soluble endoglin in plasma of *Sol-Eng* ^*+*^ and control mice. Human soluble endoglin concentrations in plasma from female (control n = 53, *Sol-Eng* ^*+*^ n = 22) (A) and male (control n = 31, *Sol-Eng* ^*+*^ n = 19) (B) mice. Data are shown as mean ± S.E.M. Mann-Whitney test, ***p≤0.001.

ELISA analysis was used to assess human soluble endoglin levels in studied mice. As shown in Fig. 1, soluble endoglin concentration in plasma was substantially higher in both female (2.477 ± 110.3 vs. 54.68 ± 16.64 ng/ml, respectively) and male (2.579 ± 115.2 vs. 37.79 ± 13.95 ng/mL, respectively) *Sol-Eng* ^*+*^ mice when compared to control mice (Fig. 1).

### Increased arterial pressure in *Sol-Eng* ^*+*^ mice

**Fig 2:**
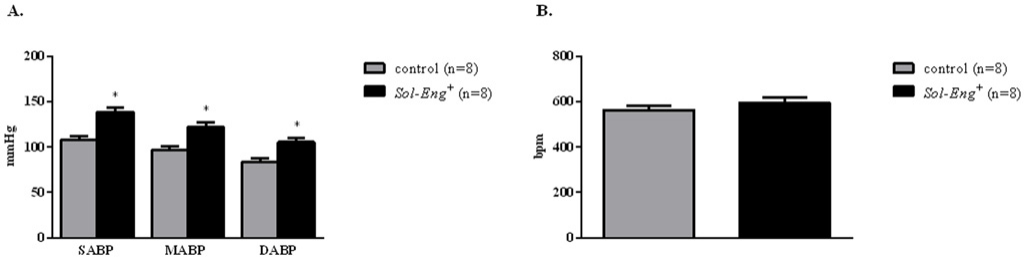
Blood pressure (A) and heart rate (B) in *Sol-Eng* ^*+*^ and control male mice assessed by telemetry. SABP: Systolic arterial blood pressure; DABP: Diastolic arterial blood pressure; MABP: mean arterial blood pressure. Data are shown as mean ± S.E.M. ANOVA and unpaired t-test with respect to control mice, *p≤0.01.

Measurements of AP by tail-cuff method (Table 1) show that systolic pressure in male and female *Sol-Eng* ^*+*^ mice is higher than that of control mice. Arterial pressure was also measured by telemetry, showing that systolic and diastolic AP in *Sol-Eng* ^*+*^ male mice was higher than that in control littermates (Fig. 2A). In the same group of animals, no significant differences in HR were observed between transgenic and control mice (Fig. 2B).

**Table 1:** Systolic arterial pressure in conscious male and female *Sol-Eng* ^*+*^ mice and controls, assessed by tail cuff.

	male		female	
	control	*Sol-Eng* ^*+*^	control	*Sol-Eng* ^*+*^
mean	110.5	121.7	108.5	122.2
S.E.M.	1.9	2.3	2.2	4.2
n	10	12	10	11
p		≤0.05		≤0.05

### Preserved NO-dependent vascular response in vivo in *Sol-Eng* ^*+*^ mice

**Fig 3:**
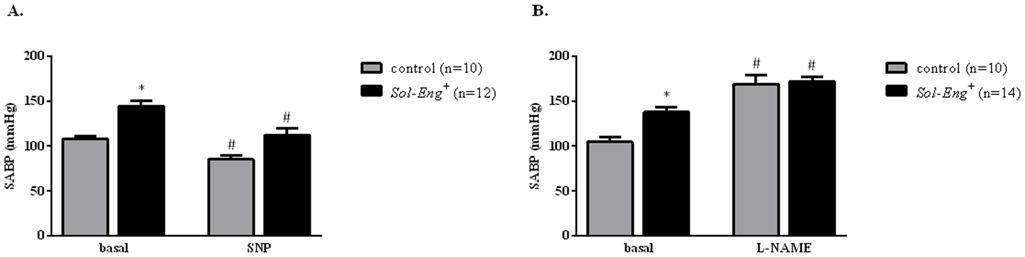
Pressure responses to agonists or antagonists of the NO-cGMP-system in *Sol-Eng* ^*+*^ and control mice. Maximal hypotensive effect of sodium nitroprusside (SNP; 2 mg/Kg b.w.) in *Sol-Eng* ^*+*^ and control mice (A). Maximal hypertensive effect of L-NAME (50 mg/Kg b.w.) in *Sol-Eng* ^*+*^ and control mice (B). Effects were measured by telemetry. SABP: Systolic blood arterial pressure. Data are shown as mean ± S.E.M. ANOVA and unpaired t-test with respect to control, *p≤0.05; ANOVA and paired t-test with respect to basal conditions, ^#^p≤0.05.

**Fig 4:**
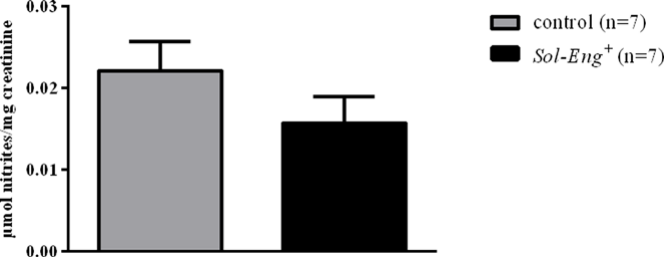
Urinary excretion of nitrites in *Sol-Eng* ^*+*^ and control mice. Urinary excretion of nitrites was measured in urine from *Sol-Eng* ^*+*^ and control mice collected in metabolic cages, and corrected by creatinine concentration. Data are shown as mean ± S.E.M. Unpaired t-test.

To test the NO-dependent function *in vivo*, we determined the response of BP to the blockade of nitric oxide (NO) synthesis and nitrite concentration in the urine. Acute administration of the NOS inhibitor L-NAME induced a sustained and similar hypertensive response in both groups of animals (Fig. 3B). Furthermore, acute administration of Ach seems to induce a similar drop of BP in *Sol-Eng* ^*+*^ mice and control animals (data not shown). However, it should be noted that the effect of acetylcholine with this experimental design was very variable, probably explained because it is very short and in many cases the hypotensive effect was artifacted by the removal of the mice from their cages to inject the drug and the consequent effects on arterial pressure, thus making difficult to correctly assess the hypotensive effect of ACh. Acute administration of sodium nitroprusside (SNP) also induced a significantly fall of BP in *Sol-Eng* ^*+*^ and control mice (Fig. 3A), with no significant differences between both groups of mice. Urinary excretion of nitrites, a stable-end product of NO metabolism, was also not statistically different in *Sol-Eng* ^*+*^ *vs*. control mice (Fig. 4).

### Preserved NO-dependent vasodilation in *ex vivo* aortic rings in *Sol-Eng* ^*+*^ mice

**Fig 5:**
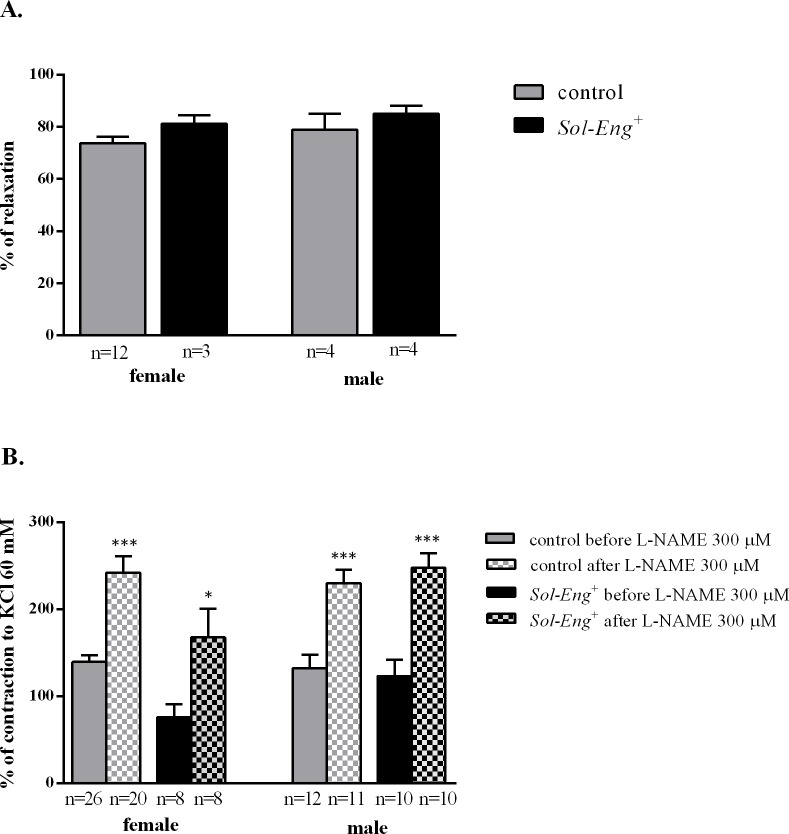
Endothelium-dependent responses in *Sol-Eng* ^*+*^ and control mice. Acetylcholine-induced relaxation in PHE or PGF2α (1 μM) pre-constricted vessels (A). Effect of L-NAME on the PHE (1 μM)-induced contraction (B). Data are shown as mean ± S.E.M. Mann-Whitney test, unpaired t-test, *p≤0.05, ***p≤0.001.

Endothelium-dependent vasodilation induced by acetylcholine (Ach) in PHE or PGF2α (1 μM) pre-contracted aorta was similar in *Sol-Eng* ^*+*^ and control female mice and also in *Sol-Eng* ^*+*^ and control male animals (81.18±3.26 *vs*. 73.65±2.46, 85.01±3.13 *vs*. 78.9±6.26, respectively) (Fig. 5A). The effect of L-NAME on PHE-induced constriction was also similar in all experimental groups (females from *Sol-Eng* ^*+*^ Δ = 92.17 and control mice Δ = 102.7; males from *Sol-Eng* ^*+*^ Δ = 97.8 and control mice Δ = 124.5) (Fig. 5B).

### No change in the expression of markers for endothelial dysfunction in aorta in *Sol-Eng* ^*+*^ mice

**Fig 6:**
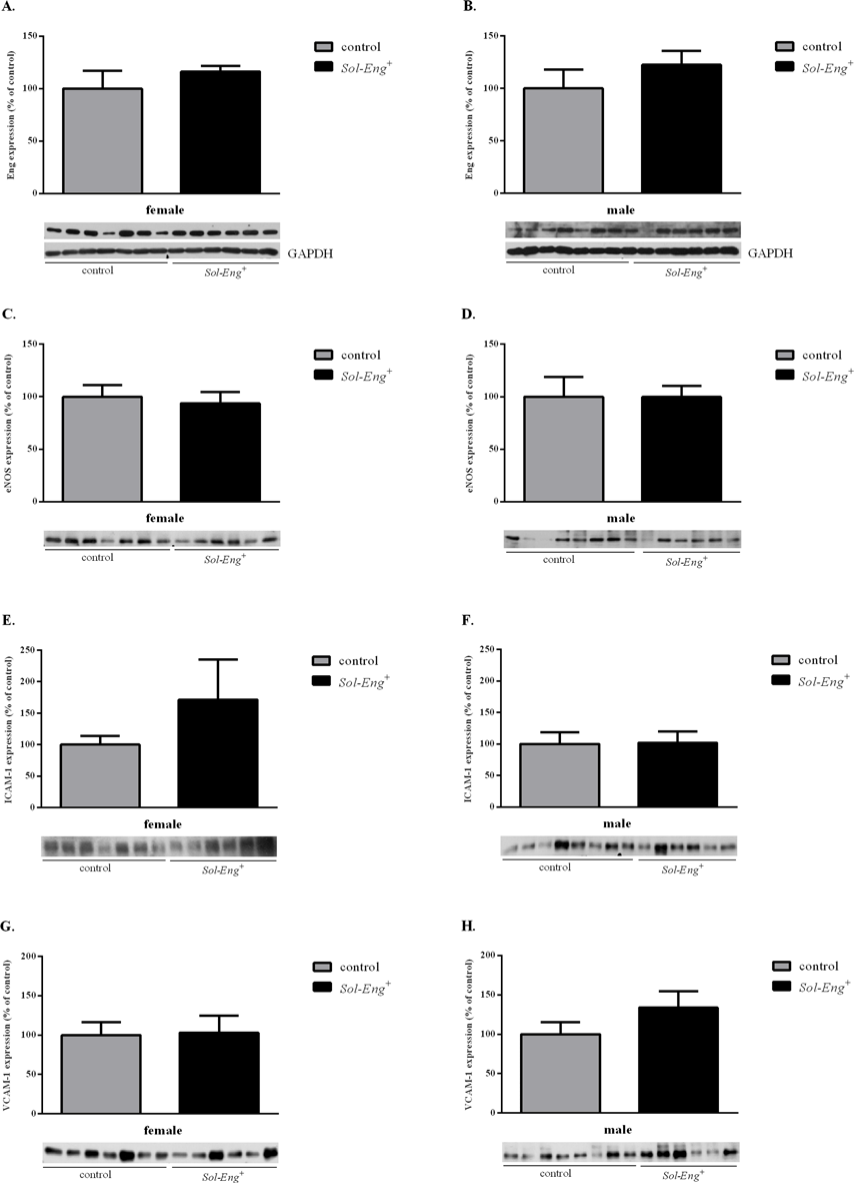
Markers of endothelial dysfunction in aorta. Expression of endoglin (A, B), eNOS (C, D), ICAM-1 (E, F) and VCAM-1 (G, H) in total protein extracts from mice aorta. Equal loading of samples was confirmed by immunodetection of GADPH (A, B). Top: densitometric analysis (control = 100%). Bottom: representative immunoblots. Data are shown as mean ± S.E.M. Mann-Whitney test, unpaired t-test.

Western blot analysis was performed in order to evaluate the changes in the aortic expression of endoglin, eNOS, ICAM-1 and VCAM-1 between *Sol-Eng* ^*+*^ and control groups. Western blot analysis revealed no significant differences in endoglin and eNOS expression in aorta between *Sol-Eng* ^*+*^ and control mice (Fig. 6A-D). In addition, no changes in the expression of cell adhesion molecules ICAM-1 and VCAM-1 were detected in *Sol-Eng* ^*+*^ mice in comparison with control animals (Fig. 6E-H).

### Alterations in vascular contractility in female *Sol-Eng* ^*+*^ mice

**Fig 7:**
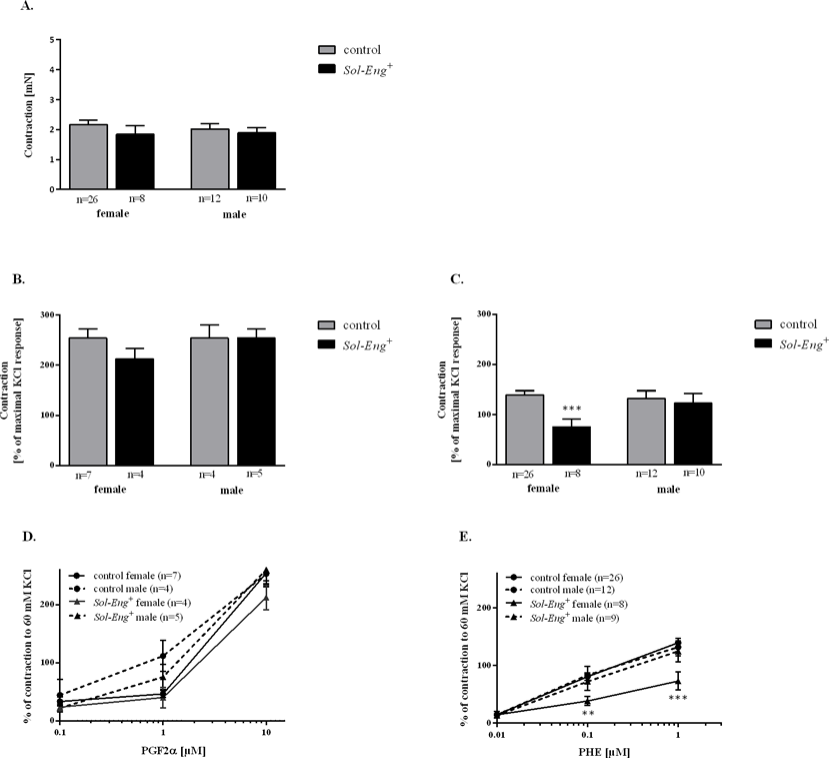
Impaired vascular contractility in female *Sol-Eng* ^*+*^ mice as compared to control mice. Maximal contraction to KCl (30 mM) (A). Maximal contraction to PGF2α (10μM) (B) and to PHE (1 μM) (C) in *Sol-Eng* ^*+*^ and control mice. Comparison of dose-response to PGF2α (D) and PHE (E) in *Sol-Eng* ^*+*^ as compared to control mice. Data are shown as mean ± S.E.M. Unpaired t-test, **p≤0.01, ***p≤0.001.

In order to evaluate a possible impact of high soluble endoglin levels on aortic function, vascular contractility was evaluated in *Sol-Eng* ^*+*^ and control mice. Receptor-independent vascular contraction induced by KCl (30 mM) was similar in aorta taken from each experimental group (control female, *Sol-Eng* ^*+*^ female, control male, *Sol-Eng* ^*+*^ male; Fig. 7A). Also, prostaglandin F2α (PGF2α)-induced vasoconstriction was not different between *Sol-Eng* ^*+*^ and control female mice (212.3±21.06 *vs*. 254.2±18.22, respectively) as well as between *Sol-Eng* ^*+*^ and control male mice (254.6±17.87 *vs*. 254.2±26.57, respectively) (Fig. 7B). In contrast, the vasoconstrictor response to phenylephrine (PHE) was significantly reduced in female *Sol-Eng* ^*+*^ mice when compared to control mice (75.53±15.39 *vs*. 139.3±7.83, respectively), while in male *Sol-Eng* ^*+*^ this response was not altered (123.2±18.98 *vs*. 132.1±15.23, respectively) (Fig. 7C). Dose-response curves for PGF2α and PHE are shown in Fig. 7D and 7E, respectively.

## DISCUSSION

A soluble form of endoglin (sEng) was demonstrated to be elevated in the sera of preeclamptic women, correlating with disease severity [3]. In addition sEng appears to contribute to the pathogenesis of pre-eclampsia by impairing binding of TGF-β1 to its receptors and downstream signaling, including effects on activation of eNOS and vasodilation, suggesting that sEng deregulate TGF-β signaling in the vasculature and may play a role in vascular dysfunction [3]. Blann et al. originally detected increased levels of sEng in hypercholesterolemic patients [6]. These authors proposed that high levels of sEng might be related to development of endothelial dysfunction in patients with hypercholesterolemia because sEng levels correlated with cholesterol levels [6]. More recently, a positive correlation between sEng plasma levels and basal glycaemia, glycated hemoglobin, endothelial dysfunction and retinopathy in patients with type II diabetes, hypertension and patients with high cardiovascular risk was observed [9]. Cui et al. showed that sEng levels and hs-CRP levels are higher in patients with plaque rupture and unstable plaques when compared with patients with stable atherosclerotic plaques [21]. Changes of sEng levels were associated with hypercholesterolemia and statin treatment in experimental atherosclerosis in mice [7,22]. Soluble endoglin was also demonstrated to increase vascular permeability [3], which is also hallmark of endothelial disturbance. All these studies, suggest that sEng might be considered as an interesting biomarker associated with hypercholesterolemia/endothelial dysfunction and atherogenesis [11].

On the other hand, a few papers showed that soluble endoglin is not only a biomarker because high levels of sEng might affect vascular endothelium function. Indeed, administration of adenoviral sEng in non-pregnant mice resulted in an increased expression of P-selectin, soluble E-selectin and VCAM-1 levels, increased number of rolling leukocytes in mesenteric venules and impaired endothelial dependent vascular autoregulation, which was related to neutralization of TGF-β signaling [12]. sEng has antiangiogenic properties, and its administration to mice induced an increase in arterial pressure [5]. As mentioned above it has been suggested that soluble endoglin interacts with TGF-β1, leading to the inhibition of binding of this protein to TGF-β receptor complex. These interactions subsequently could result in sEng-induced inhibition of TGF-β1-mediated eNOS activation in endothelial cells [1,3]. In addition, binding of soluble endoglin to BMP9 may affect the Smad1/5 signaling pathway, and, in turn, may alter endothelial function [23]. Moreover, specific binding of a mouse soluble endoglin construct (mEng^ECD^-mFc 27–581) to human BMP9 and BMP10 was demonstrated recently [24]. This study suggests that the interaction domains between endoglin and BMP9 are highly conserved between human and mouse species [24]. Therefore, it is expected that a similar binding between human endoglin and mouse BMP9 may also occur in our transgenic mice overexpressing human soluble endoglin.

In the light of these data, we hypothesized that high levels of soluble endoglin might induce endothelial dysfunction in systemic conduit vessels, such as aorta, in transgenic mice that express high levels of human soluble endoglin (*Sol-Eng* ^*+*^) [5].

*Sol-Eng* ^*+*^ mice have high plasma concentrations of human soluble endoglin within the range of 2,000–3,000 ng/mL that are far more elevated when compared to hypercholesterolemic mice with advanced atherosclerosis, where plasma concentrations of mouse soluble endoglin amounted to 2,000–3,000 pg/mL [22], which is almost 1000x less than in mice used in this study. The transgenic littermates that do not develop high levels of human soluble endoglin were used as controls in this study. It was demonstrated that *Sol-Eng* ^*+*^ mice develop a mild hypertension and proteinuria when compared to controls [5]. However, there are no data showing that high levels of soluble endoglin can induce endothelial dysfunction in aorta of these mice.

Endothelial dysfunction is characterized by the altered vascular relaxation, due to the impaired nitric oxide (NO) bioavailability that can be the consequence of either a reduced production by endothelial nitric oxide synthase (eNOS) or an increased removal by reactive oxygen species [13,25]. In addition, NO-deficiency is associated with overexpression of pro-inflammatory cell adhesion molecules like VCAM-1 and ICAM-1 in endothelial cells [26]. Moreover, decreased expression of tissue endoglin resulted in a reduced eNOS expression leading to an impaired endothelium-dependent vascular function [27].

In this study, we have observed that conscious mice overexpressing human soluble endoglin show a normal hypotensive response to acetylcholine and nitroprusside, thus demonstrating a normal-NO-mediated vascular relaxation. It should be noted that the effect of acetylcholine is very short and in many cases the hypotensive effect was artifacted by the removal of the mice from their cages to inject the drug and the consequent effects on arterial pressure, thus making difficult to correctly assess the hypotensive effect of ACh. Furthermore, although administration of L-NAME induced a lower increase of arterial pressure in *Sol-Eng* ^*+*^ than in control mice, this effect can be explained by the fact that *Sol-Eng* ^*+*^ mice already have a high pressure under basal conditions, and the reflex control of arterial pressure in conscious animals prevented a further increase. In addition, the endothelium-dependent vasodilation induced by acetylcholine in PHE or PGF2α pre-contracted aorta showed no differences between *Sol-Eng* ^*+*^ and control female and male mice. Indeed, the effect of L-NAME on PHE-induced constriction was also similar in all experimental groups, which means that high levels of sEng do not modify NO production by eNOS. In addition, Western blot analysis showed no differences in the expressions of eNOS, endoglin, VCAM-1 and ICAM-1 in aorta of both male and female *Sol-Eng* ^*+*^ and control mice. Thus, we might propose that the presence of elevated human soluble endoglin levels in plasma does not modulate the expression of membrane endoglin, cell adhesion molecules or eNOS on aortic endothelium in these mice fed with chow diet. In addition, urinary nitrite excretion, a measurement of whole body NO production, was similar in *Sol-Eng* ^*+*^ and control mice. These data clearly demonstrate that high levels of soluble endoglin alone cannot induce alterations of endothelial function in mice at least in basal normocholesterolemic conditions.

Despite the lack of changes in the expression of potential markers of endothelial dysfunction we found an impaired vascular response to PHE-induced contraction but not to PGF2α- or KCl-induced contraction in female *Sol-Eng* ^*+*^ mice, as compared to control female mice. By contrast the response in male *Sol-Eng* ^*+*^ was not altered. According to the above-mentioned results we can rule out that endothelium is involved in this impaired vascular contractility of female *Sol-Eng* ^*+*^ mice. We might speculate that specific impairment of α1 adrenergic receptors-dependent signaling in smooth muscle cells present only in female *Sol-Eng* ^*+*^ mice, in addition to the differential gender-specific hormones, might be involved. This phenomenon however requires a further investigation.

It is of interest to mention that transgenic mice with high levels of soluble endoglin on any atherosclerotic background (apoE-deficient, LDLR-deficient) are not available and thus we cannot evaluate whether high levels of soluble endoglin might contribute to endothelial dysfunction in atherosclerosis where hypercholesterolemia and inflammation are also present.

In conclusion, we demonstrate that high concentration of soluble human endoglin in plasma alone is not able to induce endothelial dysfunction in aorta of *Sol-Eng* ^*+*^ mice which, however, does not rule out a possibility that soluble endoglin might contribute to alteration of endothelial function in combination with hypercholesterolemia and/or inflammation.

## References

[1] Lopez-Novoa JM, Bernabeu C. The physiological role of endoglin in the cardiovascular system. Am J Physiol Heart Circ Physiol. 2010;299: H959–974. 10.1152/ajpheart.01251.2009 20656886

[2] Bernabeu C, Conley BA, Vary CP. Novel biochemical pathways of endoglin in vascular cell physiology. J Cell Biochem. 2007;102: 1375–1388. 17975795 10.1002/jcb.21594 PMC2199238

[3] Venkatesha S, Toporsian M, Lam C, Hanai J, Mammoto T, Kim YM, et al Soluble endoglin contributes to the pathogenesis of preeclampsia. Nat Med. 2006;12: 642–649. 16751767 10.1038/nm1429

[4] Hawinkels LJ, Kuiper P, Wiercinska E, Verspaget HW, Liu Z, Pardali E, et al Matrix metalloproteinase-14 (MT1-MMP)-mediated endoglin shedding inhibits tumor angiogenesis. Cancer Res. 2010;70: 4141–4150. 10.1158/0008-5472.CAN-09-4466 20424116

[5] Valbuena-Diez AC, Blanco FJ, Oujo B, Langa C, Gonzalez-Nunez M, Llano E, et al Oxysterol-induced soluble endoglin release and its involvement in hypertension. Circulation. 2012;126: 2612–2624. 10.1161/CIRCULATIONAHA.112.101261 23110859

[6] Blann AD, Wang JM, Wilson PB, Kumar S. Serum levels of the TGF-beta receptor are increased in atherosclerosis. Atherosclerosis. 1996;120: 221–226. 8645363 10.1016/0021-9150(95)05713-7

[7] Strasky Z, Vecerova L, Rathouska J, Slanarova M, Brcakova E, Kudlackova Z, et al Cholesterol effects on endoglin and its downstream pathways in ApoE/LDLR double knockout mice. Circ J. 2011;75: 1747–1755. 21576826 10.1253/circj.cj-10-1285

[8] Blaha M, Cermanova M, Blaha V, Jarolim P, Andrys C, Blazek M, et al Elevated serum soluble endoglin (sCD105) decreased during extracorporeal elimination therapy for familial hypercholesterolemia. Atherosclerosis. 2008;197: 264–270. 17540382 10.1016/j.atherosclerosis.2007.04.022

[9] Blazquez-Medela AM, Garcia-Ortiz L, Gomez-Marcos MA, Recio-Rodriguez JI, Sanchez-Rodriguez A, Lopez-Novoa JM, et al Increased plasma soluble endoglin levels as an indicator of cardiovascular alterations in hypertensive and diabetic patients. BMC Med. 2010;8: 86 10.1186/1741-7015-8-86 21171985 PMC3012013

[10] Ikemoto T, Hojo Y, Kondo H, Takahashi N, Hirose M, Nishimura Y, et al Plasma endoglin as a marker to predict cardiovascular events in patients with chronic coronary artery diseases. Heart Vessels. 2012;27: 344–351. 10.1007/s00380-011-0163-z 21667051

[11] Nachtigal P, Zemankova Vecerova L, Rathouska J, Strasky Z. The role of endoglin in atherosclerosis. Atherosclerosis. 2012;224: 4–11. 10.1016/j.atherosclerosis.2012.03.001 22460049

[12] Walshe TE, Dole VS, Maharaj AS, Patten IS, Wagner DD, D´Amore PA. Inhibition of VEGF or TGF-{beta} signaling activates endothelium and increases leukocyte rolling. Arterioscler Thromb Vasc Biol. 2009;29: 1185–1192. 10.1161/ATVBAHA.109.186742 19461051 PMC2775449

[13] Davignon J, Ganz P. Role of endothelial dysfunction in atherosclerosis. Circulation. 2004;109: III27–32. 15198963 10.1161/01.CIR.0000131515.03336.f8

[14] Keaney JF Jr. Atherosclerosis: from lesion formation to plaque activation and endothelial dysfunction. Mol Aspects Med. 2000;21: 99–166. 11044550 10.1016/s0098-2997(00)00005-4

[15] Najemnik C, Sinzinger H, Kritz H. Endothelial dysfunction, atherosclerosis and diabetes. Acta Med Austriaca. 1999;26: 148–153. 11512191

[16] Rossi E, Sanz-Rodriguez F, Eleno N, Duwell A, Blanco FJ, Langa C, et al Endothelial endoglin is involved in inflammation: role in leukocyte adhesion and transmigration. Blood. 2013;121: 403–415. 10.1182/blood-2012-06-435347 23074273

[17] Chamorro-Jorganes A, Grande MT, Herranz B, Jerkic M, Griera M, Gonzalez-Nunez M, et al Targeted genomic disruption of h-ras induces hypotension through a NO-cGMP-PKG pathway-dependent mechanism. Hypertension. 2010;56: 484–489. 10.1161/HYPERTENSIONAHA.110.152587 20679183

[18] Sauzeau V, Sevilla MA, Rivas-Elena JV, de Alava E, Montero MJ, Lopez-Novoa JM, et al Vav3 proto-oncogene deficiency leads to sympathetic hyperactivity and cardiovascular dysfunction. Nat Med. 2006;12: 841–845. 16767097 10.1038/nm1426 PMC1997289

[19] Tornavaca O, Pascual G, Barreiro ML, Grande MT, Carretero A, Riera M, et al Kidney androgen-regulated protein transgenic mice show hypertension and renal alterations mediated by oxidative stress. Circulation. 2009;119: 1908–1917. 10.1161/CIRCULATIONAHA.108.808543 19332469

[20] Valdivielso JM, Crespo C, Alonso JR, Martinez-Salgado C, Eleno N, Arevalo M, et al Renal ischemia in the rat stimulates glomerular nitric oxide synthesis. Am J Physiol Regul Integr Comp Physiol. 2001;280: R771–779. 11171657 10.1152/ajpregu.2001.280.3.R771

[21] Cui S, Lu SZ, Chen YD, He GX, Meng LJ, Liu JP, et al Relationship among soluble CD105, hypersensitive C-reactive protein and coronary plaque morphology: an intravascular ultrasound study. Chin Med J (Engl). 2008;121: 128–132. 18272038

[22] Rathouska J, Vecerova L, Strasky Z, Slanarova M, Brcakova E, Mullerova Z, et al Endoglin as a possible marker of atorvastatin treatment benefit in atherosclerosis. Pharmacol Res. 2011;64: 53–59. 10.1016/j.phrs.2011.03.008 21440631

[23] Gregory AL, Xu G, Sotov V, Letarte M. Review: the enigmatic role of endoglin in the placenta. Placenta. 2014;35 Suppl: S93–99. 10.1016/j.placenta.2013.10.020 24252708

[24] Castonguay R, Werner ED, Matthews RG, Presman E, Mulivor AW, Solban N, et al Soluble endoglin specifically binds bone morphogenetic proteins 9 and 10 via its orphan domain, inhibits blood vessel formation, and suppresses tumor growth. J Biol Chem. 2011;286: 30034–30046. 10.1074/jbc.M111.260133 21737454 PMC3191044

[25] Higashi Y, Noma K, Yoshizumi M, Kihara Y. Endothelial function and oxidative stress in cardiovascular diseases. Circ J. 2009;73: 411–418. 19194043 10.1253/circj.cj-08-1102

[26] Joseph-Silverstein J, Silverstein RL. Cell adhesion molecules: an overview. Cancer Invest. 1998;16: 176–182. 9541632 10.3109/07357909809050034

[27] Jerkic M, Rivas-Elena JV, Prieto M, Carron R, Sanz-Rodriguez F, Perez-Barriocanal F, et al Endoglin regulates nitric oxide-dependent vasodilatation. FASEB J. 2004;18: 609–611. 14734648 10.1096/fj.03-0197fje

